# Gender-based constraints affecting biofortified cassava production, processing and marketing among men and women adopters in Oyo and Benue States, Nigeria^☆^

**DOI:** 10.1016/j.pmpp.2018.11.007

**Published:** 2019-01

**Authors:** Olamide Olaosebikan, Bello Abdulrazaq, Durodola Owoade, Adedayo Ogunade, Olufemi Aina, Paul Ilona, Adeline Muheebwa, Béla Teeken, Peter Iluebbey, Peter Kulakow, Moshood Bakare, Elizabeth Parkes

**Affiliations:** a International Institute of Tropical Agriculture (IITA), Ibadan, Nigeria; b AUPWAE, Gender and Development Consultant, Kampala, Uganda; c Gender-Responsive Researchers Equipped for Agricultural Transformation (GREAT) RTB Fellows, Nigeria; d CIAT HarvestPlus, Nigeria

**Keywords:** Biofortified-cassava, Constraints, Gender, Nigeria

## Abstract

This study identified gender-based constraints affecting the production, processing and marketing of biofortified cassava in two states in Nigeria, using a mixed methods approach. The study identified major differences between the two study sites (Benue and Oyo). The scale of production of biofortified cassava is higher in Oyo state among adult men because of their active involvement and collaboration with research institutes within the state and the ease of transporting products to Lagos State for designated diverse markets. However, in Benue state more adult and young women are engaged in cultivation, processing and marketing business to meet up with the increased demand due to higher consumer acceptance in this region. Gender analysis revealed that lack of access to hired-labour restricted the scale of production among women in especially Oyo state. Low product price and high price of processing equipment, poor market infrastructure and middle men exploitation were constraints significantly more mentioned by women in general. Majorly, the men identified limited processing facilities/equipment as the most important constraint affecting the demand of biofortified cassava roots, while generally women were more constrained by the shortage of basic amenities and trainings that hindered their processing efficiency. The study proposes integration of gender-responsive strategies to further enhance the delivery of biofortified cassava products in Nigeria.

## Highlights

• Observed drivers of biofortified cassava production, processing and marketing varied among men and women adopters in Nigeria.
• Gender analysis show different importance on constraints experienced by men and women along the biofortified cassava value chain.
• Study informed gender responsive strategies to address the identified gendered and regional constraints.

## List of abbreviations

ADP: Agricultural development project
FG: Focus Group
FGDs: Focus Group Discussions
GREAT-RTB: Gender Responsive Researchers Equipped for Agricultural Transformation-Gender Responsive Root, Tuber and Banana Breeding course
NGOs: Non-Governmental Organisations
VAD: Vitamin A deficiency

## 1 Introduction

Micronutrient deficiencies afflict more than two billion individuals, or one in three people, globally [1]. In Nigeria, the world's largest producer and consumer of cassava products, hidden hunger in form of Vitamin A Deficiency (VAD) affects 30% of children under the age of 5 years and 20% of pregnant women [[2], [3], [4]] resulting in reduced immunity, impaired vision, and, in some cases, blindness and even death [5,6]. VAD is therefore considered a major health problem that deserves international attention [7]. These VAD-related health problems cost Africa's largest economy an estimated 1.5 billion US dollars loss in GDP [8,9]. Combating VAD especially among infants and pregnant women has led to increased efforts in breeding and delivery activities of biofortified cassava. Biofortified cassava, provides a low-cost sustainable strategy for reducing VAD leading to improved nutrition and economic livelihoods opportunities for all ages.

Cassava is a major food staple in Nigeria and especially important for the many smallholder farmers who produce by far the largest part of the cassava food products [10,11]. Cassava is a robust crop that is resistant to poor soils and drought and an important reliable resource for farmers. Biofortification of cassava through breeding for the presence of substantial levels carotenoids that can provide vitamin to the body, therefore seems a suitable and sustainable way to tackle VAD.

Studies have shown that men and women adopt new technologies at different rates [12,13] considering their diverse needs, socio-cultural context, access to available resources, unequal workloads and decision-making power [14,15]. Teeken et al. [16], shows that men and women perform different roles, which can, coupled with other factors like unequal decision making power and unequal access to agricultural resources [17,18], influence their ability or willingness to adopt new innovations. This study uses gender analysis to identify and understand the constraints faced by women and men [19] cultivating, processing and marketing biofortified cassava varieties and products in order to develop more equitable strategies for variety dissemination and utilization. We postulate that gender-responsive delivery strategies will improve food and nutrition security, and reduce poverty. Understanding specific and unique constraints for men and women in different contexts is therefore important for crop breeders, extension officers and delivery managers to work more effectively with regards to impact on productivity, income, food and nutrition security. Furthermore, gender responsive research and delivery design and implementation are appealed for by donors [[20], [21], [22], [23]] to foster equitable interventions using agricultural innovations. Donors explicitly call for an understanding of how social factors such as gender roles, age, perception and practices influences technologies benefit.

### 1.1 Biofortified (vitamin A) cassava in Nigeria

Cassava was chosen alongside maize and sweet potato as intervention crops to combat VAD in women and children [2] through biofortification breeding. According to Bouis et al.*,* [24], biofortification is the use of traditional crop breeding practices or modern biotechnology to produce micronutrient dense staple crops to reduce micronutrient deficiencies. Breeders in Nigeria have co-developed and released biofortified (vitamin A) cassava varieties with a fresh root carotenoid content in the range of 6–11 μg/g suitable for many agro-ecological conditions [25]. Biofortified cassava is increasingly been disseminated to reach more smallholder farmers in Nigeria. Dissemination is on-going through HarvestPlus delivery partners and platforms. For a biofortification program to be successful, biofortified crops need to equitably benefit both men and women along the cassava value chain [26]. While men and women smallholders both cultivate cassava, it is the women who process the cassava into food products [27] although this can vary among minorities such as immigrants who are lesser restrained by local norms of conduct [28]. Among farmers and processors in Nigeria, biofortified cassava is popularly called yellow cassava due to its inner root and product color. It serves as an alternative to vitamin A supplement drug not usually affordable and accessible to rural households when needed [29]. Biofortified cassava when consumed can provide up to 25% of daily recommended vitamin A intake [30]. Biofortified cassava varieties are being produced, processed, marketed and consumed in Nigeria and higher production, product marketing and consumption is targeted to increase widespread intake of vitamin A [31]. However, the constraining factors affecting women and men who cultivate, process and market biofortified cassava are yet to be explored and therefore subject of the study presented here.

### 1.2 Problem statement

Gender-blind agricultural research innovation and delivery activities limit rapid and sustainable impact among adopters [32]. For instance, gender influences access to agricultural knowledge and production resources such as land, labour, planting materials and training. These resources are often more accessible to men than to women [33,34]. By ignoring gender inequities, many agricultural projects fail to achieve their objective [22]. This asks for a better understanding of the influencing factors for sustaining innovation use among adopters through gender analysis. Like gender, age and region are critical factors that shapes how women and girls experience gender inequalities, express their interests, articulate their opinions and make critical choices [35]. In Nigeria, specialization and related work in cassava production, processing and marketing are gendered. While men dominate production activities like land clearing and soil tillage, women tend to dominate farm maintenance, most food processing and marketing activities [34]. Women dominate (75%) the food processing and marketing sector, while men dominate (95%) the commercial sale of cassava stems [27]. Recent studies on improved cassava varieties in Nigeria [18,35,36] are yet to adequately capture the impact of gender. This study examines the influence of gender, age, and region on the production, measured as the size of the portion of the farm attributed to biofortified cassava, and the extent of involvement in processing and marketing among adopters in Nigeria. We approached this question by examining the constraints affecting biofortified cassava production among adult (36 years and above) and young (18–35 years) men and women adopters of biofortified cassava in two major cassava cultivating regions in Nigeria. We took individual adopters of biofortified cassava as a unit of analysis, instead of households, in order to be able to identify individual experiences of women and men.

Results of this research are to contribute to the design and implementation of gender equitable strategies to upscale biofortified cassava in Nigeria.

### 1.3 Research objectives

1. To determine the scale of production of biofortified cassava among men and women adopters in selected communities in Oyo and Benue State, Nigeria
2. To identify constraining factors affecting biofortified cassava production, processing and marketing among men and women adopters in selected communities in Oyo and Benue State, Nigeria
3. To inform gender-responsive strategies and decisions towards sustenance of biofortified cassava at production, processing and marketing level among men and women adopters in Oyo and Benue State, Nigeria

## 2 Study area

This study was conducted in Oyo (South-west) and Benue (North-central) states of Nigeria. These two states belong, together with Imo and Akwa-Ibom, to the four pioneering states to have biofortified cassava stem disseminated to farmers since 2011 [36]. By 2015 about 672 communities (450,000 Nigerian households) have received the vitamin A cassava stem cuttings while 1,299,998 cassava stems have been disseminated and 245 processing centers established [37]. The target is to reach over 4,000,000 households in Nigeria in 2018.

Oyo state is one of the dominant producers of cassava [38] and its inhabitant can be classified into five groups namely: Oyo, Ibadan, Oke-Ogun, Ibarapa and Ogbomosho, also accommodating migrants from neighboring states and countries. Oyo state is home to a number of national and international agricultural research and higher educational institutions, such as Institute of Agricultural Research and Training (IAR&T), Forest Research Institute of Nigeria (FRIN), Cocoa Research Institute of Nigeria (CRIN), National Horticultural Research Institute (NIHORT), and International Institute of Tropical Agriculture (IITA) thereby creating opportunities for more farmers to participate in agricultural training, and gain access to new agricultural technologies. Although cassava is regarded as a subsistence crop of low-income families or as a “famine-reserve crop”, about 60% of the cassava output of households in the Oyo area of Nigeria is sold for processing (mostly into *gari*) while the remaining 40% is consumed at home [39,40]. Most men own and cultivate on individual plots while most of the women and also spouses to the men cultivate on plots allotted to them by their husband. However, some women own land independently through purchase, rent or inheritance as a widow and can decide on the proportion of land to cultivate to certain crops and how proceeds from their investment are utilized. Women in the study area in Oyo state prefer trading to farming. Most young women enroll as apprentice and receive skills on trading, and later become independent to start-up their own business. During the study we found that most adult and young men combined farming with other works such as teaching, farm supervisors or labour in research institute, transportation business and political activities.

Benue state is the nation's acclaimed food basket because of its fertile soil and rich agricultural produce which includes yam, rice, beans, cassava, potato, maize, soybean, sorghum, millet and cocoyam. The presence of long stretches of rivers provides the state with a potential for viable fishing, dry season farming through irrigation and with an inland water highway. Men and women cultivate on separate plots. The men own land through inheritance as male household heads/fathers or purchase or lease while the women have lands allotted to them from their spouses and in recent times, women are now able to independently own lands through purchase using personal contributions from different associations and cooperatives they belong to (tribe, religious and political) and also use the revenues from their production, processing and marketing activities. It was observed during the study, that in Benue state women are more engaged in agricultural processing and marketing activities, predominantly the food vendor business, while the men are primarily involved in production and product transportation activities.

## 3 Methods

Multi-stage sampling was used for this study. Out of ten pioneer Local Government Areas (LGAs) each in the four pioneer States: Oyo in the West, Imo in the East, Akwa-Ibom in the South and Benue in the North [27], HarvestPlus seed delivery partners confirmed and assisted in the identification of major biofortified cassava producing LGAs. Four LGAs in Oyo namely: Akinyele, Itesiwaju, Ido, and Ibarapa central and 2 LGAs in Benue namely: Otukpo and Makurdi were selected.

The state extension services office assisted in the identification of pioneer or pilot communities in each LGAs in Oyo and Benue state. In total, 12 pioneer or pilot communities were purposively selected in Oyo State and 6 communities in Benue State. Communities selected were the first to benefit from released biofortified cassava stem distributed. These communities comprised men and women adopters who still cultivated biofortified cassava during the period that the survey was conducted (2016).

Initial listing and identification of men and women farmers and processors that had adopted and were still cultivating biofortified cassava for at least 2 years was done with the assistance of extension staff of the Agricultural development project (ADP). Further selection of such adopters was complemented with snowball sampling. Transect walks through the villages were done to observe and verify cassava production, processing and marketing activities within the community.

Adopters affirmed to have obtained (directly or indirectly via Harvest plus and partners) biofortified cassava stems and had been cultivating biofortified cassava for a stipulated period of two years or more since 2012. The first biofortified cassava was released in 2011.

Therefore, adopters in this study refer to men and women engaged in firstly cultivating and often also processing and marketing of biofortified cassava roots and products for a minimum period of two years or more.

A total number of 201 respondents (125 men adopters and 76 women adopters) including farmers processors and marketers, were interviewed by trained enumerators, using a semi-structured questionnaire. The questionnaire was followed up with focus group discussions (FGDs). Direct observations and transect walks in each community were useful in validating some responses from FGDs. FGDs were conducted separately with different sex and age groups. The FGDs included a participatory exercise to formulate strategies to tackle identified constraints. The focus groups (FGs) were intended to have a minimum of 10 respondents per gender category.

Data were collected between 2nd and 23rd of December 2016. The size of the proportion of the individuals’ land cultivated with biofortified cassava was taken as a unit to measure their scale of production.

It must be mentioned that the purposive sampling method of only looking at adopters has its limitations as it reduces the number of possible respondents and diversity of information captured. Capturing information from non-adopters and farmers who discontinued cultivating biofortified cassava has been planned in a follow up study. Therefore, this research mainly provides information on the constraints experienced by adopters of biofortified cassava.

### 3.1 Description of analysis

Quantitative data entered into an Excel template was coded and analyzed using the Statistical Package for Social Analysis (SPSS) version 20. Variables were compared with demographic characteristics like sex, region and age. Descriptive and inferential statistics were computed. Frequencies, percentages, mean scores and *t*-test were used to compare and explain results.

Qualitative data was translated verbatim and transcribed. The transcripts were imported into NVivo software (Ver 10) for coding and analysis. NVivo software was used to code creating a coding tree in a sex-disaggregated manner. The analysis followed a thematic content analysis approach (Table 5) which result in a descriptive presentation of data for Fig. 3. Data was analyzed in a sex-disaggregated manner enabling comparisons for similarities and differences.

**Table 5:** Coding table used to analyse the FDG responses.

Question: In your opinion, what strategies can be put in place to increase the production, processing and marketing of biofortified cassava?			
Code	Themes-"Strategies"	Sub-themes	Keywords/concepts
1	Access to [*agricultural productive*] resources		· farm machineries
		• Inputs	· mechanized farm tools
			· modernized/new/improved farm implements
			· agro-chemicals
			· fertilizers
			· planting materials
			· mototrised farming implements
		• Loans	· borrow us money
			· give us funds/cash/capital/money
			· assist us withcredit with low or no interest rate
			· bank or government provide financial assistance
2	Access to market		· spacious and clean environment
			· motorable road networks,
		• Infrastructure	· creation of modern market for farm produce
			· product specific markets like *gari* market
			· electricity/solar
			· water supply/borehole
			· security
		• Information	· pricing information
			*· * demand for products-*gari*, abacha
3	Access to training	• Agronomic • Processing • Storage/packaging	· tell us fertilizer type and application, chemical type and application for weed management −orientation/advice/let us know/teach us how to make snacks and bread, high quality cassava flour (HQCF)
			· can make confectionaries
			· printing on nylons, nylon seal
4	Increase awareness		· cure for eye problems
		• Nutritional benefits • Empowerment benefit • Communication mediums	· profitable business
			· tell people on radio programmes/jingles
5	Variety improvement		· high yield
			· plenty roots
			· stay/store undergroung for long
			· very yellow color
6	Recognition/rewards		· prizes
			· gifts
			· certificate

## 4 Results and discussion

Results are categorized into the following sub-headings; demographic characteristics of respondents, scale of production of biofortified cassava, gender-based constraints influencing biofortified cassava production, processing and marketing as well as gender-responsive strategies to scale out biofortified cassava.

### 4.1 Demographic characteristics of respondents

Table 1 summarizes the aggregated and sex-disaggregated scores of selected demographic characteristics of respondents.

**Table 1:** Demographic characteristics of biofortified cassava farmers and processors in surveyed communities in Oyo and Benue state, Nigeria.

Aggregated Score		
Sex	Frequency (F)	Percentage (%)
Male	125	62.49
Female	76	37.81
Religion		
Islam	41	20.4
Christianity	159	79.1
Traditional	1	0.5
Age		
20–35	50	24.90
36–50	78	38.80
51–65	58	28.90
>=65	15	7.50
**Sex-disaggregated Score**	Male – F (%)	Female – F (%)
Age		
20–35	18 (36)	32 (64)
36–50	50 (64.1)	28 (35.9)
51–65	46 (79.3)	12 (20.7)
>=65	11 (73.3)	4 (26.7)
Marital status		
Married living with spouse	114 (67.6)	54 (32.1)
Married but spouse temporarily away	4 (44.4)	5 (55.5)
Divorced/separated	0 (0)	2 (10.0)
Single/Never Married	3 (37.5)	5 (62.5)
Widow/widower	1 (9.09)	10 (90.9)
Level of education		
No formal education	19 (38.8)	30(62.2)
Completed primary school	34 (58.6)	24 (41.4)
Completed Secondary school	53 (73.6)	19 (26.4)
Completed Tertiary education	15 (83.3)	3 (16.7)
Land Ownership/System of acquisition		
Owned/Inherited	73 (61.3)	46 (38.6)
Owned/Purchased	1 (20)	4 (80)
Owned/Inherited and purchased	33 (58.93)	23(41.1)
Leased/Rented	17 (85)	3 (15)
Access to Land		
Full access	110 (62.2)	67(37.8)
Partial access	19 (60.8)	4 (39.1)
Years of farming experience		
<=20 years	65 (50.4)	64 (49.6)
21–40 years	48 (80)	12 (20)
=> 41 years	10 (100)	0 (0)
Sources of Labour		
Family	6 (50)	6 (50)
Hired	64 (72.7)	24 (27.3)
Both (family and hired labour)	52 (53.6)	45 (46.4)

#### 4.1.1 Sex and age

The survey included men (62%) and women (38%) adopters of biofortified cassava, having an average age of 46.6 years, ranging from 20 to 85 years. The result of this study agrees with the findings of Arimi and Owolade [41] who put the modal age category of farmers and processors of cassava at 41–50 and 31–40 respectively. According to Orifah and Fadairo [42], cassava and cassava-based products is an energy source that can effectively meet the energy requirement of this age groups, thus, underpinning its importance.

Marital status distribution of respondents shows that 67.6% of married men who adopted biofortified cassava are living with their spouses and children while 55% of married women's spouses were temporary away for work. The results infer that married men and women had feeding responsibilities. Therefore, biofortified *gari* and other biofortified cassava products can be a cost-effective way to prevent and reduce VAD.

#### 4.1.2 Religion

20.4%, 79.1%, and 0.5% of respondents were Muslims, Christians and Traditionalist respectively. Religious institutions can be mobilized to increase sensitization on the health and nutritional benefit of biofortified cassava and its products when consumed as part of their diet.

#### 4.1.3 Land ownership/system of acquisition

Out of 125 men and 76 women adopters, 61% of men and 39% of women acquired biofortified cassava cultivated land by inheritance. Inheritance and ownership of land differ for men and women. The cultural norm and practice that women do not inherit land in households where a male child is present, is changing in recent times. Hence, women who are widows, first child or singled-child parents can now inherit land from their husbands or parent. In Nigeria, studies have shown that access to agricultural productive resources like land influences decision making capacity regarding acceptance and productivity [43,44]. Other ways of land acquisition are through purchasing and leasing.

#### 4.1.4 Source of labour

The main source of labour for biofortified cassava production was hired labour. From the one hundred and twenty-five men who adopted biofortified cassava, sixty-four (73%) engaged hired labour, while out of the seventy-six women who adopted biofortified cassava only twenty-four (27%) engaged hired labour indicating that women engage directly in production activities through their own combined with other family labour more than men who use more of hired labour. Hired labour was more available and accessible to men adopters than to women adopters in the study area. Gendered disparities in access to and ownership of resources such as land and access to labour [45,46] held true for this result.

### 4.2 Scale of production of biofortified cassava

**Fig. 1:**
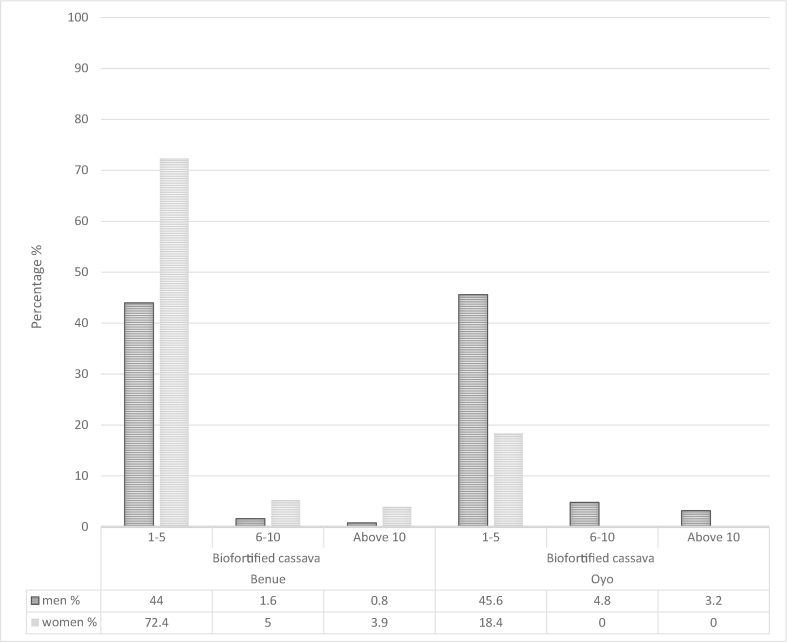
Scale of biofortified cassava production among men and women adopters in Oyo and Benue state, Nigeria. Land areas are in acres* Area of land analyzed and reported in acres.

The scale of biofortified cassava production was analyzed using individual area of land devoted to cultivation of biofortified cassava. Allocation of land area to biofortified cassava was analyzed across region/states, sex and age categories. Area of land was reported in acres. A significant difference was computed in the scale of biofortified cassava production in Oyo and Benue state, Nigeria (p = 0.013). The scale of biofortified cassava production differ among men and women in Oyo and Benue state. Fig. 1 shows that 46% of men adopters devoted between one to five acres of land to biofortified cassava cultivation while this was 18.4% for women in Oyo state, due to the men's active involvement in trainings with research institutions within the state. This also holds true for the larger individual acreage category of 6–10 acres. This result is in tandem with the summations of Arimi and Owolade, Obisesan [41,46] who both agreed that men were more involved in cassava production than women in the southwest zone of Nigeria. Arimi and Owolade [41] based the argument for men dominance in cassava production on the heavy physical labour and drudgery in farming activities which may be discouraging women from production.

In Benue state, the scale of production of biofortified cassava is higher among women adopters (72.4%) than men adopters (44%) cultivating between one to five acres of land and this also holds for the larger category of 5–10 acres. Women's higher biofortified cassava production in Benue state can be attributed to higher consumption of biofortified products especially *gari* and eba at home and in restaurants, and to the high patronage and preference for biofortified *gari* by customers. This result corroborates the assertion from Tumuhimbise et al. [47] that a crop with low patronage does not get allocated substantial acreage on the farmland. It can be stated that biofortified cassava nutritional and economic potentials are being better utilized by women than men in Benue state, due to men's high involvement in the cultivation of other crops such as yam and oil palm and because many women adopters also process and market the biofortified cassava which directly connects the production to the consumption and marketing without middlemen, while men will have to sell fresh cassava roots to processing and marketing women. The larger economic utilization of biofortified cassava by women in Benue is also reflected by the presence of women in the 6 to 10 and above 10-acre categories while in Oyo no women are present there.

**Fig. 2:**
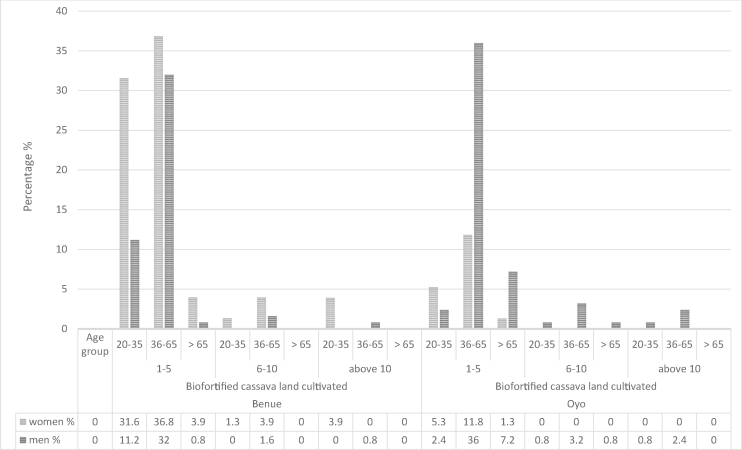
Scale of production of biofortified cassava among aged, adult and young men and women adopters in Oyo and Benue state, Nigeria. Land areas are in acres* Age group- Aged (>65), Adult (36–65) and young/youth (20–35).

In a study of economics of improved and local varieties in Nigeria, Mohammad-Lawal et al. [48] found age to be positively associated with acceptance of improved cassava varieties. This implies that the older the farmer, the higher the production scale of improved varieties. Analysis using age group (elders (>65), adult (36–65) and young/youth (20–35years)) in Fig. 2 shows that the scale of production of biofortified cassava in Benue is far higher among young women adopters (31.6%) than young men adopters (11.2%) cultivating between one to five acres of land in Benue state. In Oyo state, the scale of production of biofortified cassava is higher among adult men (36%) adopters than adult women (11.8%) adopters cultivating between one to five acres of land in Oyo state.

**Fig. 3:**
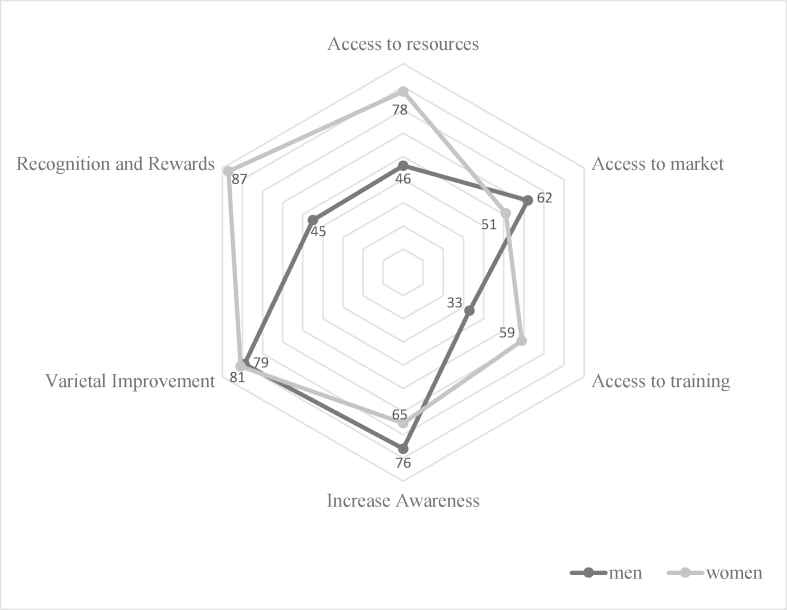
Systematic content analyzed gender responsive strategies for scaling out delivery and increasing acceptance of biofortified cassava in Nigeria.

FGDs analyses responses inferred that higher biofortified cassava production among adult and young women adopters in Benue state can be attributed to health benefit awareness programs at maternity centers targeted at women (particularly young women between 20 and 35 years). The low demand for biofortified cassava tubers by processors and low preference of consumption of its products in Oyo state is most probably related to the small scale of biofortified cassava production among women adopters and young men adopters in Oyo state who do not or do not yet have the needed profitable connections to the more elongated specific biofortified cassava markets in cities such as Lagos. The main driver of biofortified cassava production is the demand and acceptance for biofortified cassava roots and products. The larger acceptance and demand in Benue can largely be related to the practice in many places in South East Nigeria to add palm oil to the *gari*, which results into a similar yellow coloured *gari* as the biofortified cassava *gari*. This practice is much less common in the South West.

It is clear that processor and consumer demands and access to biofortified cassava fresh-roots and products create an incentive for increasing cultivation of biofortified cassava by men and women farmers respectively. This result corroborates the assertions of [49,50] that lack of marketing channels is a constraining factor to the continuous production of new cassava cultivars.

According to Tumuhimbise [47] farmers will adopt new varieties that will serve as an additional source of income or reduce expenses (production or processing cost). This is particularly important in Nigeria, where cassava is a source of food for subsistence and for income.

FGD results corroborated the descriptive results on the scale of production of biofortified cassava. Women in Benue state mentioned they are early adopters of biofortified cassava. This was affirmed by men adopters in Benue who stated that the women started the cultivation of biofortified cassava in between their husbands’ fields before the men farmers developed the interest in cultivating biofortified cassava.

The FGDs further revealed that men started cultivating biofortified cassava when they observed high yield and demand of biofortified cassava roots by the processors and acceptance of biofortified cassava products by consumers. “First of all, I observed the first and second harvest of the yellow cassava my wife planted on my field and it was very good” *Adult men FGDs, Otukpo*.

Women who adopted biofortified cassava in Benue state mentioned that they found it convenient to get involved in biofortified cassava production because of its high yield, vitamin A content and its unique yellow color as inhabitants of Benue prefer to eat *eba* and *akpu/fufu* with attractive yellow color usually derived through indigenous practice of adding palm oil during processing. Using biofortified cassava saves the costs of adding palm oil and prevents the possible rancid odour of *gari* to which palm oil is added and that is stored for a longer time. Ilona et al. [27] affirmed that the yellow color of the root and its processed products is seen as evidence of the presence of vitamin A and is liked by consumers. For instance, biofortified fufu has become a preference for fufu consumers in Akwa Ibom, while yellow *gari* (biofortified) is a growing preference for consumers in regions that traditionally consume white *gari* [27]. In contrast, in Oyo state adding palm oil to processed cassava for *gari* is not a usual practice as white coloured *gari* with a sour taste is generally preferred.

### 4.3 Gender-based constraints affecting biofortified cassava scale of production, processing and marketing

Opportunities and constraints exist along the cassava value chain flow [[51], [52], [53]]. found that the production, processing and marketing of improved varieties by value chain actors depends on how well men and women have harnessed opportunities and how well their constraints have been identified, addressed and monitored for impact. However, generalizing opportunities and constraints along the cassava value chain may reduce equitable impact of biofortified cassava for men and women. Table 2 shows *t*-test levels of significance between men and women along biofortified cassava value chain of production, processing and marketing. Some constraints are gender and region specific while others cut across both gender and region.

**Table 2:** Mean scores of constraints (on a scale of 1–5) experienced by men and women adopters of biofortified cassava at production, processing and marketing level in Oyo and Benue States, Nigeria. Constraints in each category are listed from high (more severe/important) to low (less severe/important) based on the average scores for the two sexes. For the disaggregation into states only the constraints that show significant differences between men and women are shown. Differences in mean scores between the sexes are tested for using Independent Sample T-test.

Constraints	Mean scores					
	Average Men & Women		Men	Women	t- value	*p-value*
*Production constraints*						
Poor access to loan		*3.06*	2.94	3.18	−0.87	ns
Bad road network		*3.04*	2.86	3.21	−1.28	ns
Poor mechanization		*2.90*	2.83	2.96	−0.47	ns
High cost of labour		*2.85*	2.58	3.12	−2.04	0.043**
High cost of input		*2.66*	2.40	2.91	−1.89	0.060*
High cost of weeding		*2.62*	2.37	2.87	−1.87	0.063*
Poor soil fertility		*2.36*	2.04	2.67	−2.06	0.041**
Poor access to planting materials		*2.35*	2.19	2.50	−1.07	ns
Theft/herdsmen conflict		*2.24*	2.48	2.00	1.6	ns
Lack of access to land		*2.24*	1.94	2.53	−1.87	ns
Lack of improved variety		*2.03*	1.91	2.14	−0.81	ns
Low demand for tubers		*1.81*	1.88	1.74	0.47	ns
Pest and diseases		*1.81*	1.83	1.78	0.2	ns
Unavailability of land		*1.72*	1.57	1.87	−0.94	ns
*Processing constraints*						
High cost of transportation		*2.70*	2.20	3.20	−3.44	0.001***
High cost of processing equipment		*2.55*	2.16	2.93	−2.64	0.009***
Lack of basic amenities		*2.50*	2.03	2.97	−3.33	0.001***
Unavailability of hired labour		*2.44*	1.73	3.14	−5.16	0.000***
Domestic injuries		*2.41*	1.72	3.09	−4.93	0.008*
Unavailability of market		*1.75*	1.70	1.80	−0.31	ns
Lack of training		*1.75*	1.75	1.75	0.01	ns
Insufficient tubers		*1.69*	1.66	1.72	−0.23	ns
Unorganized processing activities		*1.37*	1.37	1.36	0.04	ns
Difficulty storing product		*1.34*	1.13	1.54	−1.5	ns
*Marketing constraints*						
Poor market infrastructure		*2.70*	2.19	3.20	−3.51	0.001***
Low product price		*2.68*	2.10	3.26	−3.78	0.000***
Middle men exploitation		*2.32*	1.52	3.12	−5.35	0.000***
Unavailability of profitable market		*2.09*	2.06	2.11	−0.13	ns
Difficulty in setting prices		*1.40*	1.31	1.49	−0.62	ns
Low product quantity		*1.36*	1.38	1.33	0.16	ns
Low product quality		*1.30*	1.19	1.40	−0.12	ns
*Benue State*						
Poor market infrastructure		*3.02*	2.72	3.32	−1.79	0.08*
Domestic injuries		*2.91*	2.50	3.31	−2.34	0.02**
Low product price		*2.75*	2.43	3.06	−1.68	0.09*
Middle men exploitation		*2.60*	1.93	3.26	−3.49	0.001*
*Oyo State*						
High cost of labour		*3.07*	2.63	3.50	−1.57	ns
Low product price		*2.98*	1.82	4.14	−3.7	0.000***
Unavailability of profitable market		*2.71*	1.99	3.43	−2.15	0.04**
High cost of weeding		*2.57*	2.07	3.07	−1.82	0.07*
High cost of input		*2.48*	1.96	3.00	−1.95	0.06*
High cost of processing equipment		*2.20*	1.40	3.00	−2.7	0.008***
Lack of basic amenities		*2.16*	1.52	2.79	−2.22	0.03**
High cost of transportation		*2.06*	1.40	2.71	−2.32	0.02**
Unavailability of hired labour		*1.87*	1.10	2.64	−3.2	0.002***
Middle men exploitation		*1.83*	1.16	2.50	−0.87	0.02**

*p-value<0.10, **p-value <0.05, ****p-value <0.01, ns = not significant.

#### 4.3.1 Production constraints to biofortified cassava

Poor access to loan, poor soil fertility, high cost of weeding and high cost of labour are four major constraints significant to and affecting both men and women who adopted biofortified cassava at production level, in Oyo and Benue State. Lack or poor access to credit has been cited in several studies [54,55] as a key constraint limiting new technologies/improved seeds for increased production. Table 3, Table 4 show the gender-based constraints that affect biofortified cassava production among men and women adopters in Oyo and Benue state. Men rate poor mechanization of farm tools as significantly more important constraint in Benue state at a 0.10 level (p = 0.054), while the women in Benue state rated the ‘lack of access to more farmland’ (p = 0.093) as slightly more important. The ongoing severe conflict between herdsmen and farmers in Benue state is reflected by men as well as women rating ‘theft/herdsmen conflict’ much higher in Benue state (p = 0.0007 and p = 0.002 respectively) as significantly more important constraints within their production capacities.

**Table 3:** Aggregated mean scores of constraints (on a scale of 1–5) experienced by men biofortified cassava adopters in Oyo and Benue States, Nigeria. Constraints in each category are listed from high (more severe/important) to low (less severe/important) based on the average scores for the two states. Differences in mean scores between the states are tested for using Independent Sample T-test.

Men Constraints	Mean scores				
	Average Benue & Oyo	Benue	Oyo	t- value	p-value
*Production Constraints*					
Poor access to loan	*2.92*	2.69	3.15	−1.31	ns
Bad road network	*2.88*	3.12	2.64	1.39	ns
Poor mechanization	*2.86*	3.19	2.52	1.94	0.054*
High Labour cost	*2.58*	2.53	2.63	−0.28	ns
High cost of inputs	*2.44*	2.91	1.96	2.92	0.004***
Theft/Herdsman conflict	*2.42*	3.27	1.57	−5.01	0.0007***
High cost of weeding	*2.39*	2.71	2.07	1.88	ns
Poor access to planting materials	*2.23*	2.71	1.75	2.96	0.001***
Poor soil fertility	*2.07*	2.47	1.67	2.15	ns
Lack of access to land	*1.97*	2.45	1.49	2.5	ns
Lack of improved varieties	*1.95*	2.50	1.39	3.23	0.002***
Low demand for roots	*1.89*	2.05	1.73	0.88	0.000***
Pest and diseases	*1.86*	2.21	1.51	2.17	0.032**
Unavailability of land	*1.60*	2.02	1.18	2.23	ns
*Processing constraints*					
High cost of transportation	*2.26*	3.12	1.40	5.16	ns
High cost of processing equipment	*2.22*	3.03	1.40	4.77	ns
Lack of basic amenities	*2.07*	2.62	1.52	3.15	ns
Unavailability of hired labour	*1.78*	2.45	1.10	4.24	ns
Domestic injuries	*1.77*	2.50	1.04	4.77	ns
Lack of training	*1.76*	1.79	1.72	0.1	ns
Unavailability of market	*1.72*	1.88	1.55	0.89	ns
Insufficient roots	*1.71*	2.40	1.01	4.13	ns
Unorganized processing activities	*1.41*	1.91	0.90	2.88	0.005***
Difficulty storing products	*1.16*	1.55	0.76	2.51	0.013**
*Marketing constraints*					
Poor market infrastructure	*2.23*	2.72	1.73	2.69	0.06*
Low product price	*2.13*	2.43	1.82	1.56	ns
Unavailability of profitable market	*2.06*	2.16	1.95	0.43	ns
Middlemen exploitation	*1.55*	1.93	1.16	2.09	0.041**
Low product quantity	*1.42*	1.98	0.85	3.33	0.001***
Difficulty in setting price	*1.35*	1.76	0.93	2.46	0.015**
Low product quality	*1.23*	1.74	0.72	3.26	0.001***

*p-value<0.10, **p-value <0.05, ***p-value <0.01, ns = not significant.

**Table 4:** Aggregated mean scores of constraints (on a scale of 1–5) experienced by women biofortified cassava adopters in Oyo and Benue States, Nigeria. Constraints in each category are listed from high (more severe/important) to low (less severe/important) based on the average scores for the two states. Differences in mean scores between the states are tested for using Independent Sample T-test.

Women Constraints	Mean scores				
	Average Benue & Oyo	Benue	Oyo	t- value	p-value
*Production Constraints*					
Poor access to loan	*3.50*	3.00	4.00	−1.75	ns
Bad road network	*3.27*	3.18	3.36	−0.37	ns
High Labour cost	*3.27*	3.03	3.50	−0.94	ns
Poor mechanization	*3.12*	2.87	3.36	−0.89	ns
High cost of inputs	*2.95*	2.89	3.00	−0.21	ns
High cost of weeding	*2.95*	2.82	3.07	−0.48	ns
Theft/Herdsman conflict	*2.58*	3.50	1.66	−3.18	0.002***
Poor soil fertility	*2.39*	2.84	1.93	1.45	ns
Lack of improved varieties	*2.34*	2.03	2.64	−0.89	ns
Poor access to planting materials	*2.27*	2.65	1.89	1.36	ns
Lack of access to land	*2.19*	2.73	1.64	1.7	0.093*
Unavailability of land	*2.12*	1.73	2.50	1.14	ns
Low demand for roots	*2.09*	1.53	2.64	−1.72	ns
Pest and diseases	*1.78*	1.77	1.79	−0.02	ns
*Processing constraints*					
High transportation cost	*3.01*	3.31	2.71	1.05	ns
High cost of processing equipment	*2.96*	2.92	3.00	−0.14	ns
Unavailability of hired labour	*2.95*	3.26	2.64	1.1	ns
Lack of basic amenities	*2.91*	3.02	2.79	0.43	ns
Domestic injuries	*2.73*	3.31	2.14	1.98	0.052*
Unavailability of market	*2.13*	1.61	2.64	−1.51	ns
Lack of training	*1.96*	1.63	2.29	−1.03	ns
Difficulty storing products	*1.72*	1.44	2.00	−0.94	ns
Insufficient roots	*1.37*	1.94	0.79	1.85	0.068*
Unorganized processing activities	*1.17*	1.47	0.86	0.99	ns
*Marketing constraints*					
Low product price	*3.60*	3.06	4.14	−1.88	0.064*
Middlemen exploitation	*2.88*	3.26	2.50	1.3	ns
Unavailability of profitable market	*2.62*	1.81	3.43	−2.49	0.015**
Difficulty in setting price	*1.44*	1.52	1.36	0.27	ns
Poor market infrastructure	*1.36*	1.47	1.24	1.35	ns
Low product quality	*1.27*	1.47	1.07	0.63	ns
Low product quantity	*1.23*	1.38	1.07	0.51	ns

*p-value<0.10, **p-value <0.05, ***p-value <0.01, ns = not significant.

#### 4.3.2 Processing constraints to biofortified cassava utilization

Small and medium scale community level processing of cassava have been noted to involve some tedious, time consuming constraints. Women who adopted biofortified cassava in the sample were more likely to report that transport costs and hired labour (p = 0.001) were constraints compared to men (Table 2). High cost of transporting fresh roots from farm to processing centers/home and then to the markets due to bad road network is affecting biofortified cassava utilization potential among men and women adopters in the study area. Constraints of domestic injuries during processing activities like peeling of roots are significantly more important (p = 0.052, Table 4) for women adopters in Benue state while lack of or distant basic amenities such as ‘clean portable water’ and ‘electricity to power milling machines’ was important for both women adopters in Oyo and Benue which makes them consider hiring labour. Most times where there is access to clean portable water for processing activities such as washing and soaking of peeled tubers, it is usually distant which poses security risks for women. In communities that do not have access to borehole water, other sources like wells often do not provide enough water during the dry season and the water is difficult to fetch and bring up. This finding is in tandem with Akinnagbe [56] asserting that increased cost of transporting harvested tubers to processing centers as well as erratic climatic patterns seem to have affected optimal utilization of cassava. Processing activities with less improved/mechanized processing equipment might seem unorganized to men thereby affecting their participation. Hence, it was derived from the FGDs that men who adopted biofortified cassava prefer to sell freshly harvested roots at farm-gate or sell farm fields of unharvested roots to women. Women are much more involved in the processing and marketing of cassava products and men often consult women about the demand of fresh roots.

#### 4.3.3 Marketing constraints to biofortified cassava roots and products

Women who adopted biofortified cassava, experience lower product price for biofortified *gari* and *akpu/fufu* as more important constraint (p = 0.000), Table 2). These lower prices can be related to middlemen exploiting - which was more important to women (p = 0.000, Table 2) and particular in Benue state (p = 0.001, Table 2) product value chain, while women adopters are also experiencing ‘poor market infrastructure’ as a higher constraint than men adopters (p = 0.08, Table 2) as they are restricted to the on-farm sale of fresh roots. This also explains why men who adopted biofortified cassava in Benue state experience lack of product quality and lack of product quantity much more than men adopters in Oyo state (p = 0.0001 for both constraints, Table 3). The lower starch content (an important quality characteristic) and relative small market as compared to the market of ordinary white cassava for biofortified roots hampers men from taking up biofortified cassava production, while for women who adopted biofortified cassava, product quantity and quality is less of an issue as they market and use the biofortified cassava products as a special distinguishing health related commodity that also saves them palm oil and they also benefit from the lower prices of fresh roots for biofortified cassava enabling them to process more than they cultivate themselves. Although in Oyo state biofortified cassava has a small market, men (and mostly the adult men with a more developed network of relations) in Oyo that cultivate biofortified cassava mostly have their products taken to markets in Lagos and therefore see product quality and quantity as less of a constraint than men in Benue.

Observed shortcomings of biofortified cassava were compensated with observed benefits or opportunities. In Oyo state, adult and young men who adopted biofortified cassava, cited the need for improvement of the low starch content. The processors (mainly women adopters) who buy fresh roots from them, mentioned that the low starch content of the biofortified cassava varieties gave less product *gari* quantity. This has been the reason for low demand for biofortified cassava tubers by processors in Oyo and is therefore discouraging its production among young men in communities surveyed in Oyo state. Although Oyo state is one of the leading producers of biofortified cassava with regards to the acres of land cultivated, this production links to the commercialization of biofortified cassava products with Lagos State as one of its target markets (which also links to consumers and their preferences in Lagos that are originally from Benue state). Linking to such a specific elongated market asks for established networks and continuous production to make it profitable, reason why this production is probably more dominated by older more experienced farmers and especially men. The FGDs indicate that another reason for the consumer preference for biofortified cassava in Oyo state being comparably low at the household level could be because the major food products in the region – *amala* or *lafun* made from yam flour or cassava flour-would lose most of the carotenoid content during sun-drying if made from biofortified cassava roots.

In contrast to Oyo state, low starch content was preferred in Benue state. Respondents in Benue state regarded the low starch content as beneficial to their health conditions when consumed, this resulted in a relative high demand for biofortified cassava roots, stems and products such as *gari* and *akpu/fufu*. Hence, respondents in Benue state prefer the low starch content while respondents in Oyo state needed improvement on this. This implies that market opportunities exist for low starch content biofortified cassava varieties in the North central region (Benue state) and, if developed by breeders, high starch content yellow cassava in the South west (Oyo state).

### 4.4 Gender-responsive strategies to scale out biofortified cassava

A participatory exercise where respondents were engaged to formulate strategies to redress identified constraints was conducted in the study communities during FGDs. Fig. 3 shows gender-responsive strategies arising from roles of women as cassava processors centered around their interest in participating actively in agricultural trainings on improved agronomic practices, new processing methods and product diversification, with the condition that market days and processing activities’ time, like peeling of roots does not coincide with training time.

The recognition of women by providing rewards as early adopters and marketers of biofortified cassava products would be key to sustain its production and utilization activities in Benue state. Apart from this, gender responsive strategies in line with access to resources such as agricultural inputs and loans as well as linkage to a profitable market, through good infrastructures, where biofortified cassava tubers and products can be sold cuts across the states and affect both men and women adopters. Access is a critical dimension of technology adoption [31]. Unless the appropriate physical, economic, and information infrastructure is in place, men and women adopters of biofortified cassava may be unable to sustain their productivity and market their products. Women's gains as adopters of biofortified cassava at processing and marketing level may be limited in many contexts mostly associated with limited improved/modern processing equipment and infrastructure like good motorable roads that link processing centers and markets. Gender-responsiveness needs to go beyond increasing yield through agronomic practices as its major upscaling objective to meet specific important needs associated to men and women activities along the value chain like giving recognition/rewards, encouraging men and women's active participation in agricultural and product diversification trainings and workshop.

Women and men who have adopted biofortified cassava across sites foresee continual increase in production and a profitable market price for biofortified cassava roots and products. They noted that what would drive this change was: increased consumer's awareness and acceptance; constant and reliable access to pricing information and the availability of funds and better training on the diversification of products and accessible markets. Actualizing these drivers can increase the effectiveness of utilizing agricultural innovation (in this case biofortified cassava) in meeting the nutrition and income needs not only of farmers but also of processors, marketers and consumers along the value chain [57].

## 5 Conclusion

This study examined the scale of production of biofortified cassava among men and women adopters, and associated gender-based constraints affecting biofortified cassava production, processing and marketing activities in Oyo and Benue states. Results showed that the scale of biofortified cassava production among men and women adopters of diverse age groups and regions differ. Observed drivers of biofortified cassava production and utilization varied among men and women adopters along the value chain. It is clear that processors demand for biofortified cassava roots is the key driver of production for men adopters/farmers in the study area while consumers’ acceptance, preference and demands for biofortified cassava products, coupled with the promotion of the health benefits of biofortified (vitamin A) cassava mainly targeted at children and pregnant women, are the incentives for processors (mainly women adopters) in the study area. Gender analysis also showed differences in the severity of the constraints as experienced by men and women adopters in two different regions along the biofortified cassava value chain of production, processing and marketing. Product quantity are a constraining factor for men in Benue state as a result of the upcoming but still relatively small demand for biofortified cassava. Lack of access to hired-labour restricted the scale of production among women in especially Oyo state. Low product price and high price of processing equipment, poor market infrastructure and middle men exploitation were constraints significantly more mentioned by women in general. Men identified limited processing facilities/equipment as the most important constraint affecting the demand of biofortified cassava roots, while women in both locations were more constrained by the shortage of basic amenities and trainings that hindered their processing. Domestic injuries, time consuming root peeling of cassava and frying of *gari* is reducing women adopters processing efficiency.

Gender responsive/equitable breeding activities can be geared towards exploring the opportunity identified for lower (Benue) and higher starch (Oyo in particular) content of biofortified cassava to cater for men and women adopter's production and marketing needs as well as consumption needs in Benue state. Delivery partners should enlighten their extension services officers on gender issues and gender-based constraints along the biofortified cassava value chain and equip them with hands-on information on biofortified cassava variety names, agronomic and processing featured traits, year of release etc. This information can empower extension officers to provide gender responsive advisory service and appropriately disseminate biofortified cassava varieties that will be accepted and impactful to men and women farmers and processors in specific region/states in Nigeria.

In achieving a sustainable nationwide delivery of biofortified cassava and its nutrition security impact, health benefit awareness activities should target increasing consumer's acceptance, preference and demand for biofortified products in states/regions with low acceptance. Gender responsiveness should be applied in selecting information/communication mediums and venues suitable to identified gender and age groups like maternity centers and schools. Organization of gender responsive advocacy workshops should be done in collaboration with in the first place successful and existing biofortified cassava processors and marketers (as e.g. clearly identified in Benue) and their associations as they are most informed about the current market demand and marketing potentials. They should be linked to extension officers, and the ministry of infrastructure and ministry of agriculture and rural development in general as well as to non-governmental organisations (NGOs) for the establishment of sustainable infrastructural facilities like borehole water close to cassava processing centers will reduce drudgery and increase women biofortified cassava processing capacity and utilization efficiency. This might again encourage more young and adult men and women to seek livelihood opportunities by getting involved in biofortified cassava production, processing and marketing activities.

Gender analysis has provided the project with new options to ensure equitable delivery and impact of biofortified cassava to men and women along the value chain. Such efforts are most likely to succeed in places where biofortified cassava has already been taken up like the women processors and marketers in Benue state. This bottom-up aspect is essential. Rewarding connecting to and stimulating already existing businesses will offer a good opportunity in creating a new innovative value chain that can both empower women and men equitably. Apart from its nutritional value, biofortified cassava has a great advantage: it has a strong distinguishable appearance that can become a strong natural symbol [58] connecting modern and traditional African notions of nutrition and health, food security, aesthetics, marketing, branding and innovation.

## Declarations of interest

None.
